# Experimental Observation of Bohr’s Nonlinear Fluidic Surface Oscillation

**DOI:** 10.1038/srep19805

**Published:** 2016-01-25

**Authors:** Songky Moon, Younghoon Shin, Hojeong Kwak, Juhee Yang, Sang-Bum Lee, Soyun Kim, Kyungwon An

**Affiliations:** 1 School of Physics and Astronomy, Seoul National University, Seoul 151-747, Korea; 2 Russia Science Seoul, Korea Electrotechnology Research Institute, Seoul 121-912, Korea; 3 Korea Research Institute of Standards and Science, Daejon 305-340, Korea

## Abstract

Niels Bohr in the early stage of his career developed a nonlinear theory of fluidic surface oscillation in order to study surface tension of liquids. His theory includes the nonlinear interaction between multipolar surface oscillation modes, surpassing the linear theory of Rayleigh and Lamb. It predicts a specific normalized magnitude of 0.416*η*^2^ for an octapolar component, nonlinearly induced by a quadrupolar one with a magnitude of *η* much less than unity. No experimental confirmation on this prediction has been reported. Nonetheless, accurate determination of multipolar components is important as in optical fiber spinning, film blowing and recently in optofluidic microcavities for ray and wave chaos studies and photonics applications. Here, we report experimental verification of his theory. By using optical forward diffraction, we measured the cross-sectional boundary profiles at extreme positions of a surface-oscillating liquid column ejected from a deformed microscopic orifice. We obtained a coefficient of 0.42 ± 0.08 consistently under various experimental conditions. We also measured the resonance mode spectrum of a two-dimensional cavity formed by the cross-sectional segment of the liquid jet. The observed spectra agree well with wave calculations assuming a coefficient of 0.414 ± 0.011. Our measurements establish the first experimental observation of Bohr’s hydrodynamic theory.

Optofluidics deals with a synthetic system of optical and fluidic elements^1^,^2^,^3^, utilizing advantages of both areas^4^. As one of optofluidic examples, deformed microjet cavities are two-dimensional optical resonators supporting whispering-gallery-mode(WGM)-like cavity resonances^5^. A deformed microjet cavity is formed by a cross-sectional segment of a fluidic microjet, which undergoes surface oscillation owing to surface tension acting as a restoring force (see Fig. 1 for illustration). It provides not only a high quality factor owing to its clear and smooth surface but also high output directionality based on internal ray and wave dynamics. These features can be tuned in real time by controlling the flow rate or ejection pressure. Deformed microjet cavities are known to be a versatile platform for studying quantum chaos, prototyping highly efficient light sources as well as various photonic devices^6^,^7^,^8^,^9^,^10^.

As is often the case with tunable optofluidic components, the optical characteristics of a deformed microjet cavity is sensitive to the detailed shape of its surface boundary. Even for non-optofluidic application such as optical fiber spinning and film blowing^11^, accurate determination of multipolar components is required for consistent material processing. The cross-sectional asymmetry of a liquid jet may also affect its mechanical properties such as the oscillation period^11^,^12^,^13^ and the breakup characteristics^14^,^15^,^16^. For optical cavity applications, in particular, it is necessary to measure the boundary profile of this cavity as accurately as possible. However, it is generally not easy to measure the accurate shape of a fluidic object. Liquid surface exhibits specular reflection and thus make the conventional optical triangulation technique inapplicable^17^. Moreover, the continuous columnar shape of the microjet makes top-view imaging of its boundary simply impossible.

It may seem that these difficulties in measurement might be easily compensated for by a theoretical approach. It is particularly because there exists a well-established hydrodynamic theory to describe the surface oscillation of a liquid jet, mostly owing to Lord Rayleigh and Sir Lamb^12^,^18^. However, the surface shape predicted by this theory is accurate only when the oscillating amplitude is infinitesimally small because the theory is based on the first-order approximation. It was Niels Bohr at his early career who first extended the surface oscillation theory by including nonlinear interactions^13^. The extended theory and accompanying surface tension measurement of his own based on the lowest-order surface oscillation period enabled him to win a gold medal in an academic contest of the royal Danish academy. However, the surface oscillation period does not reveal the multipolar surface-oscillation amplitudes, which is the hallmark of Bohr’s theory. So far, direct experimental verification of the multipolar surface oscillation amplitudes has not been reported.

In this paper, we present two independent experimental studies to determine the multipolar surface-oscillation amplitudes in the boundary profile of a deformed microjet cavity. One is a non-destructive surface reconstruction experiment based on optical forward diffraction. The other is the spectroscopy of the cavity resonances compared with wave calculation results for various cavity boundary profiles. We found the results of both studies exhibit good agreement with Bohr’s prediction in nanometer-level accuracy. Our work thus marks the first experimental verification of Bohr’s hydrodynamic theory. Moreover, precise knowledge of our microjet cavity boundary allows us to predict optical and dynamical properties of cavity resonances in advance. Quality factors, output directionality and intermode interactions can be predicted beforehand to guide actual experiments for various photonics and optofluidic applications such as biosensors^19^, optical filters and mode switchers^20^.

To begin with, let us discuss the differences between the two theories when they are applied to a deformed microjet. The microjet used in this study is operated under the following conditions. It is ejected vertically from a deformed orifice, which can be decomposed into quadrupolar and octapolar components mostly as shown in Fig. 1c. The mean radius of the orifice is varied in a range of 11–15 *μ*m. The vertical velocity *v*_*z*_ of the jet is held fixed, in a range of *v*_*z*_ ~ 10–20 m/s, by regulating the ejection pressure so that the surface shape becomes stationary. The room temperature is held constant in a range of 15–25 °C. More details about the jet setup are described in Methods and ref. 5.

According to the linear theory of Rayleigh and Lamb^12^,^18^, the *m*th mode of surface oscillation can be expressed in the cylindrical coordinates as


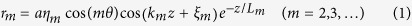


where *a* is the mean radius, *η*_*m*_ is the relative amplitude, *k*_*m*_ ≡ 2*π*/Λ_*m*_ is the wave vector in *z* direction with Λ_*m*_ the oscillation wavelength, *ξ*_*m*_ is the initial phase at the orifice and *L*_*m*_ is the decay length. The parameters Λ_*m*_ and *L*_*m*_ from the linear theory are given by following formulae:


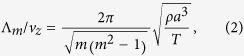



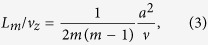


where *T*,*ρ* and *ν* are the surface tension, the density and the kinematic viscosity of the liquid, respectively, and *v*_*z*_ is the speed of the jet. The quantities Λ_*m*_/*v*_*z*_ and *L*_*m*_/*v*_*z*_ are the oscillation period and the decay time, respectively.

When the jet material is ethanol and the temperature is 20 °C, *T* = 2.23 × 10^−2^ N/m, *ρ* = 789 kg/m^3^, and *ν* = 1.52 × 10^−6 ^m^2^/s. Thus in this case we obtain the period of quadrupolar oscillation Λ_2_/*v*_*z*_ = 28.0 *μ*s from Eq. (2) and the decay time *L*_2_/*v*_*z*_ = 37.0 *μ*s from Eq. (3). The Reynolds number, estimated as 2*av*_*z*_/*ν* in our case, is about 200, so putting our experimental regime away from hydrodynamic instability^21^. Comparing the quadrupolar and octapolar modes, we find *L*_2_/*L*_4_ = 6 and 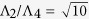. That means, during one period of the quadrupolar oscillation (Λ_2_ = 300–600 *μ*m), the quadrupolar amplitude is halved whereas the octapolar amplitude decays by 2 orders of magnitude (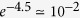). Therefore, after one quadrupolar period, the octapolar mode should become hardly noticeable according to the linear theory, and thus we expect that the cross-sectional shape of the jet at extreme positions D*n* with *n* > 2 as marked in Fig. 1d would be approximated as


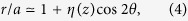


where *η*(*z*) is identified as the eccentricity 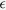 of the cross-sectional shape, given by the relation


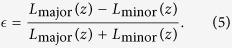


where *L*_major_(*z*) and *L*_minor_(*z*) are the lengths of the major and minor axes at the observed extreme position, respectively.

The result of Bohr’s nonlinear calculation, on the other hand, for the lowest-order oscillation of a nonviscous liquid jet can be written as^13^,


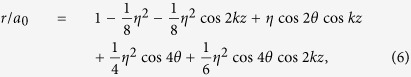


where *k* is the wave vector in the propagation direction, and *a*_0_ is the mean radius when *η* = 0. Considering only extreme positions, and taking the major axis of the shape as the *θ* = 0 axis in the *r*-*θ* coordinates, Eq. (6) is simplified as


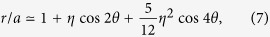


where *a* = *a*_0_(1 − *η*^2^/4) is the mean radius for the nonvanishing *η*, coming from flux conservation for a fixed jet speed. The main distinction of Eq. (7) from Eq. (4) of the linear theory is the existence of an octapolar component. Moreover, its amplitude is given by the square of the quadrupolar deformation with a specific coefficient of 5/12. In Bohr’s theory, the octapolar component is induced by the quadrupolar one because of the nonlinear coupling between them.

It is instructive to compare Eq. (7) with the boundary profile of an ellipse, which is a regular-shape object with a similar deformation. The profile of an ellipse can be decomposed in multipoles as


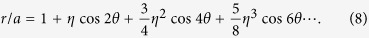


Note that the ellipse has a different coefficient of 3/4 = 0.75 in front of *η*^2^ instead of 5/12 = 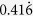 in Eq. (7) 22. In order to verify Bohr’s prediction, one then needs an experimental technique which can not only detect a small octapole component but also can resolve its coefficient.

## Results

### Surface reconstruction

The first experimental method we employ to measure the surface shape of the deformed microjet is a direct surface mapping technique. This technique, developed in our previous study^23^, is based on forward shadow scattering (see Methods). We repeatedly performed the shape measurement under various initial conditions such as different ejection pressure, types of solvent and mean radii at several extreme positions D*n* (*n* > 2) on the jet.

All boundary profiles we reconstructed exhibit 2-fold axial symmetry as shown in the inset of Fig. 2. The profiles are dominated by a quadrupolar component, and the second largest component is an octapole component. Note that the magnitude of the octapole component in almost all cases cannot be explained by the linear theory. For example, the profile in the inset of Fig. 2 is obtained at an ejection pressure of 2.6 bar with *r* = 15 *μ*m and *v*_*z*_ = 16 m/s at D3, which is located at 860 *μ*m (corresponding to about 2Λ_2_) from the orifice. The relative magnitudes of the quadrupolar and octapolar components there are 23.9(±0.3)% and 2.6(±0.2)%, respectively. The magnitudes of the higher poles are smaller than 0.1%, which is within the error range of the present technique. If the linear theory were correct, the observed magnitude of the octapole component should come from an initial relative magnitude exceeding 15000%, which is simply impossible.

The failure of the linear theory motivates us to apply Bohr’s theory to the observed data. If Bohr’s prediction is correct, there would be a relation between the magnitude *η*_1_ of the quadrupolar component and the magnitude *η*_2_ of the octapolar component as


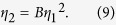


The proportionality constant *B*, which we affectionately call Bohr factor or simply B factor, should be equal to 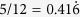. Figure 2 summarizes the B factors extracted from the observed boundary profiles under different experimental conditions such as mean radii, types of solvent and eccentricities. Interestingly, they are centered around a common value *B* = 0.42 ± 0.08, if we require *B* to be a constant, despite the different experimental conditions. The boundary profile can then be described by the following empirical equation:


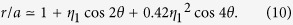


Note that the observed coefficient 0.42 is consistent with Bohr’s prediction of 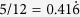 within the experimental error.

### Spectroscopy and wave calculation

Our second experimental method is spectroscopic observation of cavity-modified fluorescence (CMF) spectrum. A small *z* segment of a few micron thickness of the liquid column at an extreme position acts as a two-dimensional dielectric cavity for the optical wave. The boundary of the cavity is given by the cross-sectional (perpendicular to the direction of the jet advance) shape of the jet. The liquid contains dye molecules. When the small *z* segment of the jet column is excited by a pump laser as illustrated in Fig. 6c, the fluorescence from dye molecules is enhanced at the cavity resonances as shown in Fig. 3a. This enhancement is the cavity quantum electrodynamics effect^24^. In this CMF spectrum from the microjet cavity, we typically observe 4–6 groups of high-*Q* (quality factor) cavity resonances or modes. Each mode group is a sequence of resonances with a well-defined free spectral range (FSR).

Once the wavelengths (*λ*) of the resonances are identified, they are converted into a *normalized* size parameter *X* = 2*πna*/(*n*_0_*λ*), where *n* is the wavelength- and temperature-dependent refractive index of the cavity medium (ethanol) and *n*_0_ = 1.361, the value of *n* at 610 nm and 20 °C. With this normalisation, it is then easy to compare the positions of resonances experimentally observed with those found by numerical calculations since the latter are given in the size parameter 2*πna*/*λ*.

The CMF spectrum in Fig. 3a was measured with cavity eccentricity 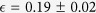, which was obtained by using Eq. (5) with the measured *L*_major_ and *L*_minor_ values from the microscope image. The CMF spectrum was taken in a spectral range from 540 nm to 660 nm and all cavity modes were identified. Their relative normalized size parameters (labeled as X Gap) are then displayed in Fig. 3b with respect to reference normalized size parameters. The reference normalized size parameters (displayed on the horizontal axis) are given by an arithmetic sequence with a mean FSR of all mode groups. The spectral resolution was 0.007 nm at *λ* = 579.1 nm, corresponding to a resolution of 0.002 in *X* for a radius around 14 *μ*m. The diagram obtained in this way as in Fig. 3b is called the ‘mode evolution’ diagram^9^. It clearly shows intermode interactions, *i.e.*, crossing and avoided crossing of modes.

These observed modes are then compared with those by numerical wave calculation. The wavelength and linewidth of a cavity resonance are obtained by solving the Maxwell equations for a cylindrical dielectric medium whose 2-D boundary profile is given by Eq. (4) (quadrupole deformation only) with 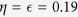. We use the boundary element method for the wave calculation^25^.

For visual comparison, the mode evolution diagram obtained from the wave calculation is displayed in Fig. 3c. If Eq. (4) gives a correct description of the cross-sectional shape of the jet, the experimental and theoretical mode evolution diagrams should match each other. Instead, we find a big discrepancy. In the experiment we observe 5 distinct high-*Q* mode groups whereas we find only 4 such groups in the wave calculation. Moreover, the way the modes interact with each other shows a complete discrepancy: the relative spectral positions of all resonance-modes are quite different in both cases.

One may suspect that the uncertainty in the eccentricity might be responsible for such a big discrepancy. We find, however, that in order to see 5 high-*Q* mode groups in the wave calculation the eccentricity has to be less than 0.14, which is beyond our experimental uncertainty. Moreover, even with such a low eccentricity the mode evolution diagram from the wave calculation look completely different from the experimental mode evolution diagram. We then have to conclude that the cross-sectional profile of the jet is not given by Eq. (4).

Is then the observed CMF spectra consistent with Bohr’s nonlinear theory? In order to address this question, we repeatedly changed the trial boundary shape and calculated the mode evolution diagram. We then found that all of the experimentally obtained mode evolution diagrams are in good agreement with the wave calculation results when the boundary profile is close to the empirical formula Eq. (10). For example, the experimental mode evolution diagram in Fig. 3b is in good agreement with the wave calculation result for the boundary profile given by Eq. (10) with *η*_1_ = 0.185 ± 0.003. This *η*_1_ value corresponds to an eccentricity 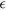 of 0.182 ± 0.003, which is consistent with the measured eccentricity of 0.19 ± 0.01 for our liquid jet within the experimental error. Besides, five more mode diagrams are presented in Fig. 4 for a wide range of *η*_1_.

One can evaluate the tolerance in the B factor value of 0.42 by examining the difference between the observed mode evolution diagram and the calculated one as a function of *B* around the empirical value of 0.42. For quantitative analysis, we pay attention to the avoided crossing gap between mode groups 2 and 4, which are denoted by G2 and G4, respectively, in Figs 4 and 5. The G2-G4 avoided crossing gap for *η*_1_ = 0.185 is calculated from the wave equation for various B factor values from 0.37 to 0.45 in Fig. 5a. We find that the experimental avoided crossing gap is well fit by the B factor value of 0.414 ± 0.011. This is also supported in Fig. 5b, where both wave calculations with *B* = 0.41 and 0.42 well reproduce the experimental mode evolution diagram for *η*_1_ = 0.185.

## Discussion

Both of our experimental studies indicate that Bohr’s description of the surface shape of an oscillating jet is correct one whereas the linear theory of Rayleigh and Lamb is not. Between the two experimental methods, the CMF spectroscopy combined with the wave calculation takes much longer time than the surface reconstruction method but yields the narrower error range.

Nevertheless, the difference between Bohr’s prediction and that of the linear theory is a small octapole term. One may then wonder how such a small shape difference can affect the cavity resonances so significantly as shown in Fig. 3b,c. Considering *particle motion* in a deformed billiard is helpful to answer this question. In quantum chaos, it is a well-established fact that there exists a close connection between a classical trajectory of a particle (in our case, ray trajectory) and a wave-mechanical solution (cavity resonances) for a given billiard shape^26^,^27^. It is demonstrated in Supplementary Note 1 that a long-time particle or ray dynamics in a quadrupolar billiard is very sensitive to a shape perturbation. If experiments are performed with low-*Q* cavities, which is often the case with etched semiconductor microcavities, long-time dynamics hardly survive to affect the cavity resonances much. On the other hand, if we deal with high-*Q* resonances, such as in dynamical tunneling and mode evolution, long-time dynamics becomes important and thus a small error in the boundary profile can lead to a considerable difference^10^,^28^,^29^,^30^,^31^. By this reason, the spectroscopy of high-*Q* resonance modes can serve as a very sensitive measure of the boundary shape of the 2-D optical billiard or the cross-sectional shape of our liquid jet.

Though the Bohr’s theory is more accurate than the linear theory, it is, too, based on many approximations. Particularly the *z*-dependence is treated quite simply there: the surface motion is regarded as a two-dimensional oscillation and its time dependence is simply replaced by the z-dependence with an assumption that the fluid velocity is a constant. This treatment is by no means rigorous by the following reasons. First, under gravity, the fluid velocity is not constant. If the jet is ejected vertically upward like in our experimental setup, the velocity slows down. Second, the conservation of the flux dictates the velocity has to change periodically. The sectional area given by Eq. (7) is 
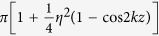
, having *z* dependence. Therefore, the velocity also has to change as *z* in order keep the flux conserved.

If we limit ourselves to the region where the surface oscillations have not decayed sufficiently, *i.e.*, at several oscillation wavelengths away from the orifice, the change of velocity due to gravity is estimated to be negligibly small for our jet (less than 10^−4^ of the initial velocity). The velocity change due to the flux conservation, however, amounts to 1% of the initial velocity, in the same order of magnitude as the octapole component in the typical boundary profile. This is not small enough to be neglected, and it may affect the boundary profile. Consequently, we cannot completely rule out the possibility that the agreement between the experimental result Eq. (10) and Bohr’s analysis Eq. (7) might be accidental under the particular conditions that the experiment was done.

In order to resolve this problem, we have extended Bohr’s inviscid 2-D analysis into 3-D by including the effects of gravity and flux conservation. We have then obtained the coefficient attached to *η*^2^*cos*4*θ* as


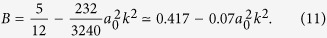


The detailed derivation is presented in Supplementary Note 2 – it should be pointed out that the hydrodynamic master equations treated with Poincaré-Lindstedt method^32^ fail to include all of the nonlinear terms required in Bohr’s nonlinear theory. The term containing (*a*_0_*k*)^2^ is very small in our experiment: 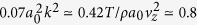 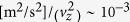 considering that the velocity *v*_*z*_ is typically 10–20 m/s. Experimentally, such a small correction is obscured by our experimental error. Therefore, our calculation confirms that the nonlinear analysis by N. Bohr is adequate for describing the motion of our liquid microjet. However, if *a*_0_*k* is comparable to or larger than unity such as in the case of garden hose or home faucet, the adequateness of Bohr’s analysis is not guaranteed and we expect that our extension should be applied.

## Methods

### The deformed microjet

**Figure 1:**
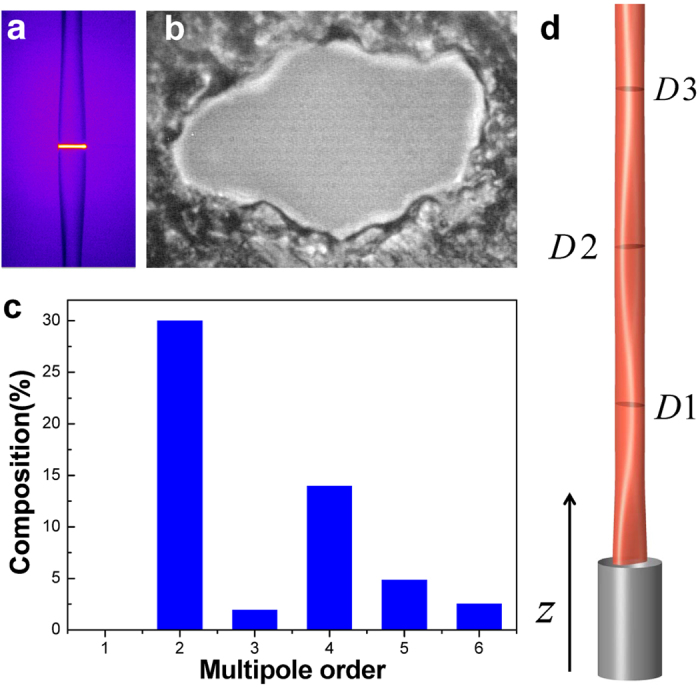
Deformed microjet cavity based on fluidic surface oscillation. (**a**) Microscope image of the liquid column of a deformed microjet seen from the side. Mean radius is 15 *μ*m. The bright region of about 3 *μ*m thickness is the segment excited by a pump laser beam to form a two-dimensional microcavity. Image from Kwak *et al.*^33^. (**b**) Microscope image of an orifice for the deformed microjet. The orifice is filled with liquid to obtain a clear image. (**c**) Multipolar decomposition of the boundary profile of the orifice of (**b**). The origin is chosen so as to make the dipolar component vanish. In this decomposition, quadrupolar (denoted by 2) and octapolar (denoted by 4) components are dominant. (**d**) A 3-D model of the deformed microjet. The pattern is stationary while the liquid moves in *z* direction. Extreme positions of surface oscillation with stationary cross sections are indicated as D1, D2, D3, and so on.

**Figure 2:**
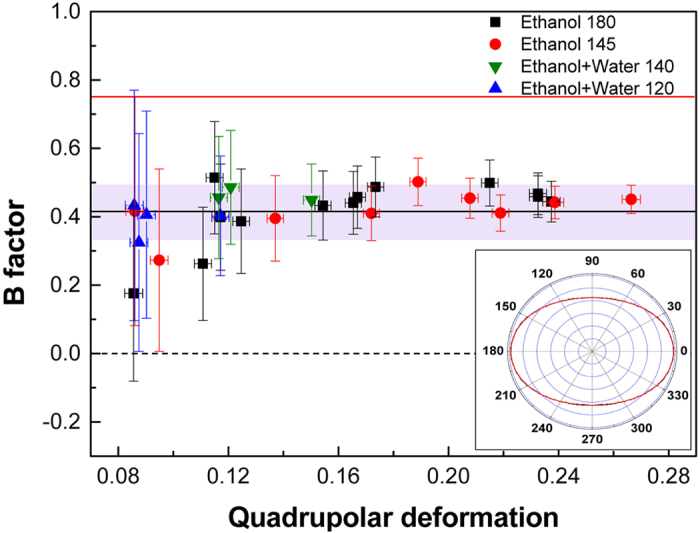
B factor values for various deformations and materials. The black-filled squares and red-filled circles are data of ethanol, while the blue- and green-filled triangles are that of ethanol-water 50:50 mixture. Numbers are the normalized size parameter *X*. The averaged B factor (black line) is clearly distinguished from the B factor for an ellipse (red line at *B* = 0.75) and that for a quadrupole (dotted line at *B* = 0). The fitting error is indicated as a shaded region. Inset: An example of the reconstructed profile. The experimental conditions are indicated in the text.

**Figure 3:**
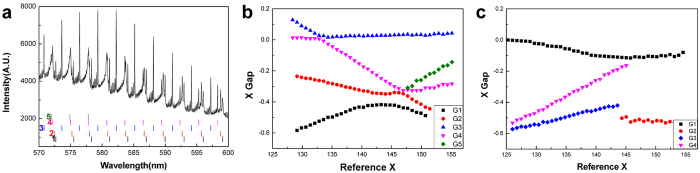
CMF spectra inconsistent with the linear theory. (**a**) The observed CMF spectrum and identification of five groups (labeled as 1, 2, …, 5) of cavity modes. The spectrum is measured at D4 with a jet-ejection pressure of 1.408 bar. The jet medium is ethanol doped with Rhodamine590 dye at a concentration of 0.05 mM/L. The mean radius of the jet is *a* = 14 ± 1 *μ*m. Positions of the cavity modes are marked by vertical bars colored differently for each mode group. (**b**) Experimentally observed mode evolution diagram when 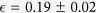. (**c**) Mode evolution diagram obtained by the wave calculation for the boundary profile given by Eq. (4) with 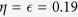. Legends G1, G2, etc. denote cavity mode groups.

**Figure 4:**
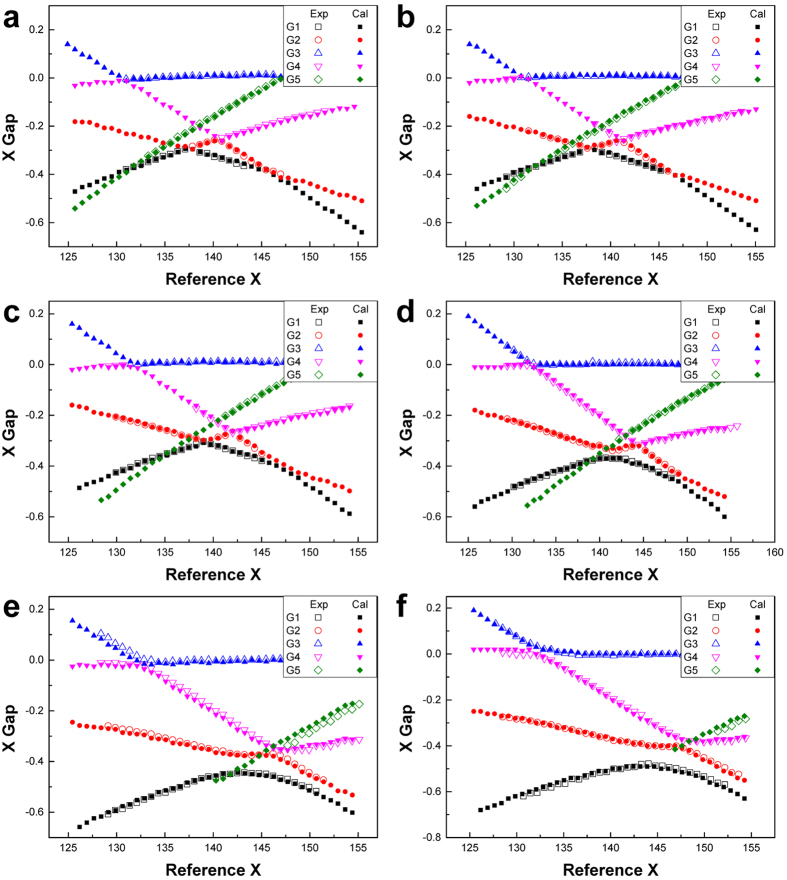
Matching the mode evolution diagrams between experiment and wave calculations. Experimental conditions are expressed as ‘(jet pressure in bar, extreme position D*n*, jet temperature in °C)’. Jet mean radius *a* for each case is determined as a fitting parameter. Legends ‘Exp’ and ‘Cal’ stand for experiment and wave calculation, respectively. (**a**) Experiment at (1.250, D5, 16.9) and the wave calculation with *η*_1_ = 0.095 and *a* = 13.79 *μ*m. (**b**) Experiment at (1.402, D5, 16.9) and the wave calculation with *η*_1_ = 0.105 and *a* = 13.71 *μ*m. (**c**) Experiment at (1.326, D5, 21.4) and the wave calculation with *η*_1_ = 0.130 and *a* = 13.64 *μ*m. (**d**) Experiment at (1.188, D4, 20.3) and the wave calculation with *η*_1_ = 0.160 and *a* = 13.61 *μ*m. (**e**) Experiment at (1.408, D4, 19.5) and the wave calculation with *η*_1_ = 0.185 and *a* = 13.55 *μ*m. (**f** ) Experiment at (1.536, D4, 19.1) and the wave calculation with *η*_1_ = 0.200 and *a* = 13.50 *μ*m. The data in (**e**) is the same as the data in Fig. 3c.

**Figure 5:**
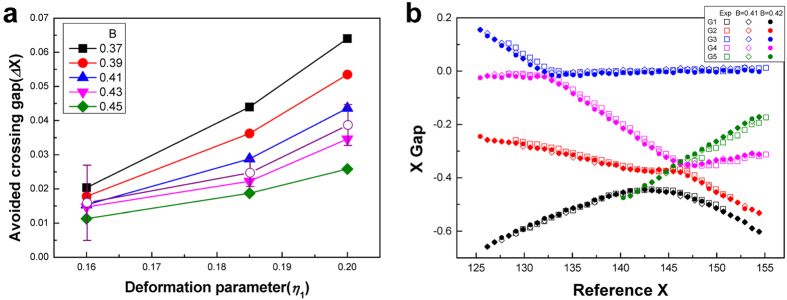
Tolerance in the B factor. (**a**) G2 – G4 avoided crossing gaps from the experiment (open circles with error bars) is compared with those from the wave calculation with the B factor varied from 0.37 to 0.45 for *η*_1_ = 0.160, 0.185, 0.200. (**b**) Mode evolution diagrams for *B* = 0.41 and *B* = 0.42 for *η*_1_ = 0.19.

A liquid stream from a non-circular orifice shows semi-periodic surface oscillations due to surface tension as a restoring force. Such oscillations are often encountered in our daily lives as in coffee poured from a pot or water from a faucet. The deformed boundary shape of our microjet cavity is also due to this surface oscillation. In our setting, a liquid jet is vertically ejected from a near-elliptical orifice^5^ as shown in Fig. 1d. The major and minor axes of the horizontal cross-sections are repeatedly exchanged as we move along the vertical axis. This phenomenon is known as ‘axis switching’^15^. Moreover, the amplitude of surface oscillation decays and thus the cross-sectional shape would gradually converge to a circle. From the microscope images of the jet like Fig. 1a, we can easily identify the directions of major and minor axes of cross-sectional shape. Moreover, the axis-switching is also clearly observed. The average period of the axis switching for the jet is 270 ± 10 *μ*m in Fig. 1a. A small *z* segment of a few micron thickness of the liquid column at an extreme position acts as a two-dimensional dielectric cavity for the optical wave. The boundary of the cavity is given by the cross-sectional (perpendicular to the direction of the jet advance) shape of the jet. The liquid contains dye molecules as the gain material.

### Surface reconstruction experiments

In our previous studies, we developed an optical technique to map out the cross-sectional profile of an opaque cylindrical object from its forward diffraction patterns with a resolution of 0.1% of the mean diameter of the object^23^. This technique is particularly useful for fluidic columns like a liquid microjet, where the conventional electronic imaging techniques such as scanning electron microscopy or scanning tunneling microscopy cannot be applied. Its working principle is as follows. When a plane wave is incident to an opaque cylindrical object, it can be shown that the angular separation ΔΘ between two adjacent local minima of the diffracted-wave intensity distribution near the forward direction is given by the following simple relation.


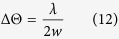


where 2*w* is the projected full width of the object in the forward direction and *λ* is the wavelength of the incident plane wave. We can then reconstruct the cross-sectional profile of the object by measuring *w* at various projection angles, covering a range of at least 180° for an object of two-fold axial symmetry. Variation of the projection angle is conveniently done by rotating the cylindrical object on a rotatable stage for a fixed incident plane wave. The details of the boundary profile reconstruction procedure is reported in ref. 23.

**Figure 6:**
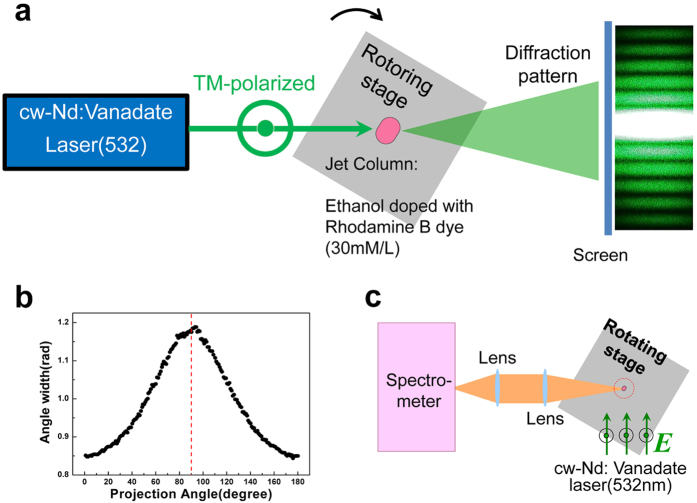
Experimental methods. (**a**) Top view of our experimental setup and a real image of the diffraction pattern. (**b**) An example of the angle width function. (**c**) Experimental setup for CMF spectroscopy (top view). The incident laser is polarized in the direction parallel to the jet column.

In our experiment depicted in Fig. 6a, a liquid jet assembly is mounted on a rotary stage and the jet vertically ejected is illuminated by a cw laser beam at 532 nm (or 514 nm in some cases), which is focused by a cylindrical lens with a focal length of 7 cm down to a vertical waist of 3 *μ*m on the jet. The diffraction pattern of the laser beam by the jet column is then examined on a screen located at 2.4 m from the jet. The mean radius of the jet column is about 15 *μ*m while the horizontal waist of the laser beam is 2.3 mm, fully covering the jet column horizontally. About 5–6 local minima lying within 10° from the forward direction are used to obtain a mean value of ΔΘ for a given rotation angle *θ* of the jet assembly as shown in Fig. 6b. The typical value of the resulting ΔΘ is about 1 degree.

The requirement of opaqueness is satisfied by doping the liquid jet with dye molecules until the optical density due to absorption for a laser beam going through the diameter exceeds 6.9. For this, Rhodamine 590 dye, whose absorption cross section *σ* at *λ* = 532 nm is 3 × 10^−21 ^m^2^, is dissolved in ethanol at a molar density of 23 mM/L. The boundary profile measurement is repeated for various initial conditions such as different ejection pressure, types of solvent and mean radii at several critical positions D*n* (*n* > 2) on the jet.

## Additional Information

**How to cite this article**: Moon, S. *et al.* Experimental Observation of Bohr’s Nonlinear Fluidic Surface Oscillation. *Sci. Rep.* **6**, 19805; doi: 10.1038/srep19805 (2016).

## Supplementary Material

Supplementary Information
