# Supplementary material for: Experimental Observation of Bohr’s Nonlinear Fluidic Surface Oscillation
Source: Sci Rep. 2016 Jan 25;6:19805. doi: 10.1038/srep19805 (PMC4726443; doi:10.1038/srep19805)
Supplement: Supplementary Information [file srep19805-s1.pdf]

# Experimental Observation of Bohr's Nonlinear Fluidic Surface Oscillation

Songky Moon,[1,*] Younghoon Shin,[1] Hojeong Kwak,[1,†] Juhee Yang,[2] Sang-Bum Lee,[3] Soyun Kim,[1] and Kyungwon An[1,‡]

[1]*School of Physics and Astronomy, Seoul National University, Seoul 151-747, Korea*

[2]*Russia Science Seoul, Korea Electrotechnology Research Institute, Seoul 121-912, Korea*

[3]*Korea Research Institute of Standards and Science, Daejon 305-340, Korea*

(Dated: July 25, 2015)

*Present Address: Faculty of Liberal Education, Seoul National University, Seoul 151-742, Korea

†Present Address: Samsung Electronics Company, Hwaseong 445-330, Korea

‡Electronic address: kwan@phys.snu.ac.kr

## SUPPLEMENTARY INFORMATION

### I. EFFECT OF A SMALL PERTURBATION IN RAY DYNAMICS

It is known that non-regular boundary shapes such as quadrupole can induce chaotic dynamics in the classical particle description and also result in the interactions among resonance modes in the wave description. In addition, it is known that even quite a small perturbation can change the particle dynamics significantly in a partially chaotic system. Considering the close connection between classical dynamics and wave solutions, we expect a small perturbation of a right type may explain the above discrepancy with the spectroscopic observations.

A regular billiard which has a profile similar to a quadrupole is an elliptical billiard, whose boundary can be expressed as an infinite series of multipolar components as Eq. (8) in the main text. The shape difference between a pure quadrupole and the ellipse with the

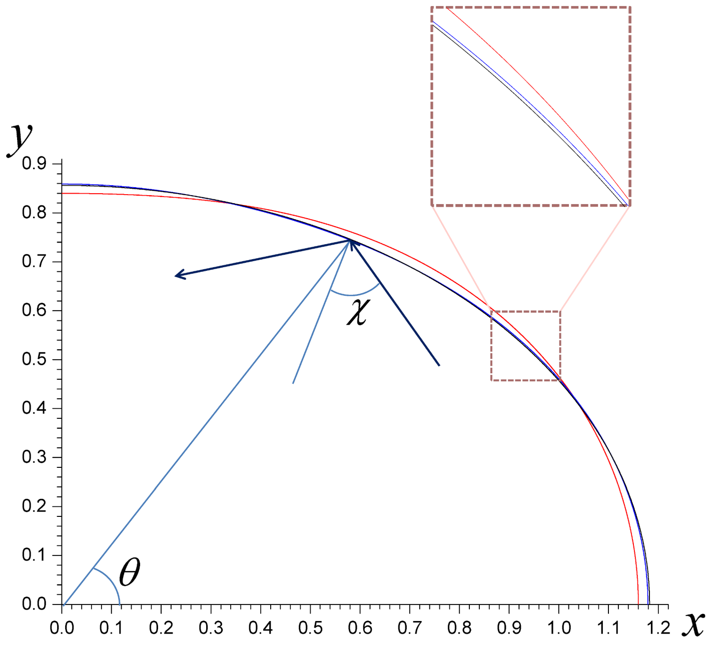


FIG. S1: **Comparison of elliptical, semi-elliptical and quadrupolar billiards.** All of the elliptical (black), semi-elliptical (blue) and quadrupolar (red) billiards have the same eccentricity $\epsilon = 0.16$ and the same enclosed area. When a particle is reflected off the boundary, its angular position is denoted by $\theta$ with an reflection (or incident) angle $\chi$.

same eccentricity is quite small, amounting to at most 1/100 of the mean diameter $2a$ in the deformation range of $0 < \eta < 0.2$ as seen in Fig. S1. Beyond $\eta = 0.2$, the convexity of quadrupole is broken like peanut due to the contraction along the minor axis. However, internal ray dynamics in the elliptical billiard are quite different from those of the pure quadrupolar billiard. It is because a small initial difference in particle trajectories can grow exponentially later on in the presence of chaos.

Classical particle dynamics is conveniently analyzed in Poincaré surface of sections (PSOS), which is commonly used to visualize the particle motion in a 2-dimensional billiard. PSOS is a phase-space diagram which is drawn iteratively in terms of reflection position ($\theta$) and reflection angle ($\chi$) of a particle upon bouncing off the boundary. Regular or quasi-periodic orbits are represented by concentration of dots around localized substructures such as islands or curves. Chaotic motion has no such concentration of dots, rather randomly but uniformly filling some regions called chaotic sea in the phase space.

Figure S2a shows the PSOS of a pure quadrupole while Fig. S2b shows that of an ellipse, both drawn in terms of $\theta$ and $\sin\chi$ for $\epsilon = 0.16$. Almost the whole PSOS in Fig. S2a is chaotic while the PSOS in Fig. S2b consists of many periodic and quasi periodic orbits without chaos. Since it is practically impossible to fabricate a billiard with a perfectly elliptical or perfectly quadrupolar boundary, it is also meaningful to consider the PSOS of a truncated semi-ellipse with the same eccentricity for comparison as in Fig. S2c. The profile of a semi-elliptical billiard is obtained by truncating the expansion in Eq.(8) beyond the second order of $\eta$. Though chaotic sea is present in considerable regions of the phase space, we still have curve-like or island-like structures corresponding to regular trajectories. From these

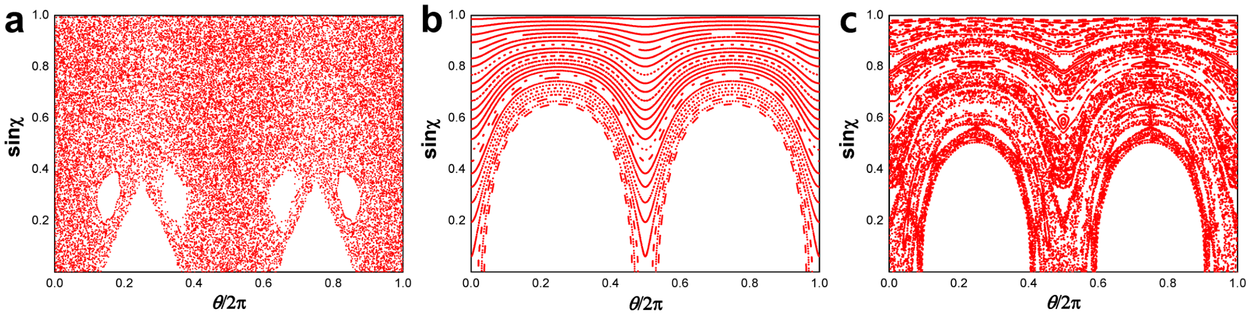


FIG. S2: **Poincaré surface of sections for various billiards.** (a) A pure quadrupole given by $1 + 0.16\cos 2\theta$, (b) an ellipse and (c) a semi-ellipse, both with an eccentricity $\epsilon = 0.16$.

considerations, we find that even quite a small difference in the boundary profile can change the particle dynamics significantly. In particular, degradation of the regular dynamics is striking when an ellipse is replaced with a semi-ellipse with the same eccentricity.

## II. IMPROVED NONLINEAR ANALYSIS

In order to derive the expression for the $B$ factor more rigorously, we have modified the nonlinear calculation of Niels Bohr to include the $z$-dependence up to the second order of $\eta$. We include the viscous decay in terms of an exponential factor multiplied to an inviscid surface oscillation mode. Then the surface motion can be treated by introducing the potential $\phi$ for the velocity $\mathbf{v}$, satisfying $\nabla\phi = \mathbf{v}$.

Master equations of motion derived from the Bernoulli's principle under the stationary condition are then written as follows in the cylindrical coordinates $(r, \theta, z)$:

$$\nabla^2\phi = 0, \tag{S1}$$

$$\frac{T}{\rho a}\left(\frac{a}{R_1} + \frac{a}{R_2}\right) + \frac{1}{2}\left(\frac{\partial\phi}{\partial r}\right)^2 + \frac{1}{2r^2}\left(\frac{\partial\phi}{\partial\theta}\right)^2 + \frac{1}{2}\left(\frac{\partial\phi}{\partial z}\right)^2 = C, \tag{S2}$$

$$\frac{\partial\phi}{\partial r} - \frac{1}{r^2}\frac{\partial\phi}{\partial\theta}\frac{\partial r}{\partial\theta} - \frac{\partial\phi}{\partial z}\frac{\partial r}{\partial z} = 0, \tag{S3}$$

where $C$ is a constant and $R_1$ and $R_2$ represent two principal radii of curvature of the jet surface. Equations (S1)-(S3) account for the irrationality of the fluid, the Bernoulli's principle and the condition for unbroken surface, respectively. The sum of the reciprocals of the principal radii is just the trace of the shape operator in differential geometry:

$$\begin{aligned}\frac{a_0}{R_1} + \frac{a_0}{R_2} = &\left[-r^3\frac{\partial^2 r}{\partial z^2} + 2r\frac{\partial r}{\partial\theta}\frac{\partial r}{\partial z}\frac{\partial^2 r}{\partial\theta\partial z}\right.\\ &-r\frac{\partial^2 r}{\partial\theta^2}\left(\frac{\partial r}{\partial z}\right)^2 - r\left(\frac{\partial r}{\partial\theta}\right)^2\frac{\partial^2 r}{\partial z^2}\\ &\left.-r^2\left(\frac{\partial r}{\partial z}\right)^2 + r^2 + 2\left(\frac{\partial r}{\partial\theta}\right)^2 - r\frac{\partial^2 r}{\partial\theta^2}\right]\\ &\left[r^2 + \left(\frac{\partial r}{\partial\theta}\right)^2 + r^2\left(\frac{\partial r}{\partial z}\right)^2\right]^{-\frac{3}{2}}.\end{aligned} \tag{S4}$$

The surface motion is decomposed by the orders of magnitude in $\eta$:

$$r/a_0 = F^{(0)} + \eta F^{(1)} + \frac{1}{2}\eta^2 F^{(2)}, \tag{S5}$$

$$\phi = \phi^{(0)} + \eta\phi^{(1)} + \frac{1}{2}\eta^2\phi^{(2)}. \tag{S6}$$

Then Eq. S4 becomes

$$\begin{aligned}\frac{a_0}{R_1} + \frac{a_0}{R_2} \;=\; & 1 - \eta\left[F^{(1)} + \frac{\partial^2 F^{(1)}}{\partial\theta^2} + a_0^2\frac{\partial^2 F^{(1)}}{\partial z^2}\right] \\ & -\frac{1}{2}\eta^2\left[F^{(2)} + \frac{\partial^2 F^{(2)}}{\partial\theta^2} - 2\left(F^{(1)}\right)^2\right. \\ & -4F^{(1)}\frac{\partial^2 F^{(1)}}{\partial\theta^2} - \left(\frac{\partial F^{(1)}}{\partial\theta}\right)^2 + a_0^2\left(\frac{F^{(1)}}{\partial z}\right)^2 \\ & \left.+a_0^2\frac{\partial^2 F^{(2)}}{\partial z^2}\right].\end{aligned} \tag{S7}$$

The zeroth motion corresponds to an upward cylindrical flow of a circular cross section. The gravitation causes the vertical velocity to decrease as the height: $v^2 = {v_z}^2 - 2gz$, where $g$ is the gravitational acceleration and $v_z$ is the initial velocity. At the same time the radius has to increase to conserve the flux. Therefore, $F^{(0)}$ and $\phi^{(0)}$ are given by

$$F^{(0)} = 1 + \frac{gz}{2{v_z}^2}, \tag{S8}$$

$$\phi^{(0)} = \frac{gr^2}{4v_z} + v_z z - \frac{g}{2v_z}z^2. \tag{S9}$$

The $r$-dependence of the velocity potential is required to satisfy the Laplace equation. With these expressions substituted in Eq. (S2), we can identify $C = T/(\rho a_0) + v_z^2/2 - gz$. However, the terms containing the gravitational acceleration $g$ are too small to make noticeable contribution. They will be thus neglected from now on.

The motion corresponding to the first-order terms is quite similar to the motion from the linear analysis. The master equations are:

$$\nabla^2\phi^{(1)} = 0, \tag{S10}$$

$$-\frac{T}{\rho a_0}\left[F^{(1)} + \frac{\partial^2 F^{(1)}}{\partial\theta^2} + {a_0}^2\frac{\partial^2 F^{(1)}}{\partial z^2}\right] + v_z\frac{\partial\phi^{(1)}}{\partial z} = 0, \tag{S11}$$

$$\frac{\partial\phi^{(1)}}{\partial r} - a_0 v_z\frac{\partial F^{(1)}}{\partial z} = 0. \tag{S12}$$

A solution of the Laplace equation in the cylindrical coordinates can be written as a linear sum of Bessel's functions $J_n$. Therefore above equations are solved by substituting

$$\begin{aligned} F^{(1)} &= \cos n\theta \cos kz \\ \phi^{(1)} &= M_n J_n(ikr) \cos n\theta \sin kz \end{aligned} \tag{S13}$$

where $n = 1, 2, 3, \ldots$ and $M_n$ is a coefficient to be determined. Equations (S11) and (S12) then yield

$$M_n = -\frac{T}{\rho a k v_z J_n(ika_0)}(n^2 + k^2 {a_0}^2 - 1) = \frac{i a_0 v_z}{J'_n(ika_0)}. \tag{S14}$$

The factor $(ka_0)$ in the Bessel's function is noticeable. It is interpreted as an index number representing the ratio of the mean radius to the $z$-axial wavelength. A circular cylinder is the case when $ka_0 \to 0$.

Equation (S14) is further simplified when $ka_0 \ll 1$. Using the approximated expression for the Bessel's function, the above relation can be approximated as

$$(ka_0)^2 = \frac{T}{\rho {a_0}^3 v_z^2} n(n^2-1)\left[1 + \frac{3n-1}{2n(n^2-1)}(ka_0)^2\right]. \tag{S15}$$

or by solving for $ka_0$ we get

$$ka \simeq \sqrt{n(n^2-1)}\left(\frac{T}{\rho a_0 v_z^2}\right)^{1/2}\left[1 + \frac{3n-1}{4}\left(\frac{T}{\rho a_0 v_z^2}\right)\right] \tag{S16}$$

When the term proportional to $(ka_0)^2$ is negligible, the equation is reduced to the wave number formula derived in the linear analysis, Eq. (2) in the main text. When $n = 1$ (dipolar motion), Eq. (S16) is satisfied if and only if $k = 0$. The dipolar motion corresponds to a constant translation of the center of cross-sectional mass. It can be neglected as far as surface oscillation is concerned. Therefore, the quadrupolar ($n = 2$) motion is the lowest oscillatory motion.

Besides the Laplace equation, the second ordered equations are more complicated.

$$\begin{aligned} &- \frac{T}{\rho a_0}\left(F^{(2)} + \frac{\partial^2 F^{(2)}}{\partial \theta^2} + {a_0}^2 \frac{\partial^2 F^{(2)}}{\partial z^2}\right) + v_z \frac{\partial \phi^{(2)}}{\partial z} \\ &+ \frac{T}{\rho a_0}\left[-2\left(F^{(1)}\right)^2 + \left(\frac{\partial F^{(1)}}{\partial \theta}\right)^2 - 4{a_0}^2 F^{(1)} \frac{\partial^2 F^{(1)}}{\partial z^2}\right. \\ &\left. -{a_0}^2\left(\frac{\partial F^{(1)}}{\partial z}\right)^2\right] + \left(\frac{\partial \phi^{(1)}}{\partial r}\right)^2 + \frac{1}{{a_0}^2}\left(\frac{\partial \phi^{(1)}}{\partial \theta}\right)^2 \\ &+ \left(\frac{\partial \phi^{(1)}}{\partial z}\right)^2 + 4 v_z F^{(1)} \frac{\partial \phi^{(1)}}{\partial z} + 2 a_0 v_z F^{(1)} \frac{\partial^2 \phi^{(1)}}{\partial z \partial r} = 0, \end{aligned} \tag{S17}$$

$$
\begin{aligned}
\frac{\partial \phi^{(2)}}{\partial r} &- a_0 v_z \frac{\partial F^{(2)}}{\partial z} + 4F^{(1)}\frac{\partial \phi^{(1)}}{\partial r} - \frac{2}{a}\frac{\partial \phi^{(1)}}{\partial \theta}\frac{\partial F^{(1)}}{\partial \theta} \\
&- 4a_0 v_z F^{(1)}\frac{\partial F^{(1)}}{\partial z} - 2a_0\frac{\partial \phi^{(1)}}{\partial z}\frac{\partial F^{(1)}}{\partial z} \\
&+ 2a_0 F^{(1)}\frac{\partial^2 \phi^{(1)}}{\partial r^2} = 0. \qquad \text{(S18)}
\end{aligned}
$$

By substituting the expressions in Eqs. (S13) and (S16) into $F^{(1)}$ and $\phi^{(1)}$, Eqs. (S17) and (S18) become

$$
\begin{aligned}
&- \left(F^{(2)} + \frac{\partial^2 F^{(2)}}{\partial \theta^2} + a^2\frac{\partial^2 F^{(2)}}{\partial z^2}\right) \\
&+ \frac{T}{\rho a_0}\left[\frac{(n^2-1)^2}{{v_z}^2} + \frac{2(n^2-1){a_0}^2k^2}{{v_z}^2}\right]\cos^2 n\theta \cos^2 kz \\
&+ \frac{\rho a_0 v_z}{T}\frac{\partial \phi^{(2)}}{\partial z} + \left[n^2\sin^2 n\theta\cos^2 kz\right. \\
&\quad \left.- (2-4{a_0}^2k^2)\cos^2 n\theta\cos^2 kz - {a_0}^2k^2\cos^2 n\theta\sin^2 kz\right] \\
&+ \frac{\rho a_0}{T}{a_0}^2k^2{v_z}^2\cos^2 n\theta\sin^2 kz + \frac{T}{\rho a_0}\left[\frac{n^2(n^2-1)^2}{{a_0}^2k^2{v_z}^2}\right. \\
&\quad \left. + \frac{2n^2(n^2-1)}{{v_z}^2} + \frac{n^2{a_0}^2k^2}{{v_z}^2}\right]\sin^2 n\theta\sin^2 kz \\
&- 4(n^2-1+{a_0}^2k^2)\cos^2 n\theta\cos^2 kz \\
&- \frac{2\rho a_0}{T}{a_0}^2k^2{v_z}^2\cos^2 n\theta\cos^2 kz = 0, \qquad \text{(S19)}
\end{aligned}
$$

$$
\begin{aligned}
&\frac{\partial \phi^{(2)}}{\partial r} - a_0 v_z\frac{\partial F^{(2)}}{\partial z} \\
&+ \frac{T}{\rho a_0}\frac{1}{a_0 k v_z}n^2(n^2-1+{a_0}^2k^2)\sin^2 n\theta\sin 2kz \\
&- \frac{T}{\rho a_0}\frac{a_0 k}{v_z}n^2(n^2-1+{a_0}^2k^2)\cos^2 n\theta\sin 2kz = 0. \qquad \text{(S20)}
\end{aligned}
$$

The terms comparable to or smaller than ${a_0}^4k^4T/(\rho a_0)$ have been neglected since the sum of them would contribute the final result by no more than 0.01%.

Now we are ready to evaluate the second ordered motion $F^{(2)}$. The velocity potential must satisfies both Laplace equation and Eq. (S20). Therefore, $\phi^{(2)}$ and $F^{(2)}$ can be written as

$$\phi^{(2)} = J_0(i2kr)\gamma_0(z) + J_{2n}(i2kr)\cos 2n\theta\gamma_{2n}(z),$$
$$F^{(2)} = \delta_0(z) + \cos 2n\theta\delta_{2n}(z). \tag{S21}$$

The function $\delta_0(z) = \sin^2 kz$ is related to the area conservation. Carrying on the calculation further yields the expression for $\delta_{2n}(z)$.

$$\begin{aligned}\delta_{2n}(z) &= \frac{2n^3+5n^2-2n-2}{4(4n^2-1)} + \frac{n^2+n+1}{4n(4n^2-1)}{a_0}^2k^2 \\ &+ \left[\frac{-2n^3+7n^2+2n-4}{4(2n^2+1)}\right. \\ &+ \left.\frac{-40n^5+22n^4+23n^3+17n^2+11n+1}{4n(2n+1)(2n^2+1)^2}{a_0}^2k^2\right] \\ &\times\ \cos 2kz.\end{aligned} \tag{S22}$$

The expression for $B_{n=2}$, the coefficient attached to $\eta^2\cos 4\theta$, is then obtained from $\delta_4(z)$ with $n = 2$ (octapole component) and $\cos 2kz = 1$ (nodal and anti-nodal positions):

$$\begin{aligned}B_{n=2} &= \frac{1}{2}\,\delta_4(z)|_{\cos 2kz=1} = \frac{5}{12} - \frac{232}{3240}{a_0}^2k^2 \\ &\simeq 0.417 - 0.07{a_0}^2k^2.\end{aligned} \tag{S23}$$

which is Eq. (13) quoted in Discussion in the main text.
